# Trends in Cardiovascular Disease Prevalence by Income Level in the United States

**DOI:** 10.1001/jamanetworkopen.2020.18150

**Published:** 2020-09-25

**Authors:** Salma M. Abdalla, Shui Yu, Sandro Galea

**Affiliations:** 1 Department of Epidemiology, Boston University School of Public Health, Boston, Massachusetts; 2 Boston University School of Public Health, Boston, Massachusetts

## Abstract

**Importance:**

Income disparity between persons with the most resources (the top 20% of earners) and the remainder of the population in the United States has dramatically widened over the past few decades. Given the well-established association between income and health, this increasing income gap may provide insights into the dynamics of cardiovascular disease (CVD) burden among adults in the US.

**Objective:**

To quantify the contribution of people in the highest-resources group and the remainder of the population to the burden of CVD, and to estimate the trends in the prevalence of CVD for the 2 groups in the United States from 1999 to 2016.

**Design, Setting, and Participants:**

This serial cross-sectional analysis used nationally representative data from 9 cycles of the National Health and Nutrition Examination Survey (NHANES) between 1999 and 2016. Survey participants were adults 20 years or older. Statistical analysis of the data was conducted in December 2019.

**Main Outcomes and Measures:**

Age-standardized prevalence of CVD was calculated using the 2010 census estimates. Participants were stratified by income group using the NHANES income to poverty ratio, which was converted into a binary variable: a ratio of 5 or greater for the highest-resources group and a ratio of less than 5 for the remainder of the population.

**Results:**

This analysis included 44 986 participants. Among the 7926 participants in the highest-resources group, 3290 (50.3%) were in the 40- to 59-year age group and 4094 (51.9%) were men. Among the 37 060 participants in the remainder of the population, 10 840 (34.1%) were in the 40- to 59-year age group and 19 470 (53.2%) were women. The age-standardized prevalence of CVD decreased in the highest-resources group during the study period (1999-2016): angina from 3.4% (n = 24) to 0.3% (n = 5), heart attack from 3.2% (n = 24) to 1.4% (n = 19), congestive heart failure (CHF) from 1.2% (n = 11) to 0.5% (n = 7), and stroke from 1.1% to 1.0% (n = 8). In the remainder of the population, the prevalence of angina decreased from 3.3% (n = 131) in 1999 to 2.6% (n = 118) in 2016 and heart attack from 4.0% (n = 160) in 1999 to 3.6% (n = 201) in 2016. Conversely, an increase was observed in the prevalence of CHF from 2.6% (n = 123) in 1999 to 2.8% (n = 176) in 2016 and stroke from 2.9% (n = 152) in 1999 to 3.2% (n = 178) in 2016. Over time, the odds of reporting angina (odds ratio [OR], 0.80; 95% CI, 0.73-0.87; *P* < .001), heart attack (OR, 0.91; 95% CI, 0.86-0.97; *P* = .003), and CHF (OR, 0.90; 95% CI, 0.82-0.99; *P* = .03) decreased among those in the highest-resources group, while there was no significant change in the odds of reporting stroke (OR, 0.97; 95% CI, 0.90-1.05; *P* = .43). Among the remainder of the population, the odds of reporting angina (OR, 0.95; 95% CI, 0.92-0.99; *P* < .05) and heart attack (OR, 0.99; 95% CI, 0.97-1.02; *P* = .06) decreased over time. Conversely, there was no statistically significant change in the odds of reporting CHF (OR, 1.02; 95% CI, 1.00-1.05; *P* = .08) and stroke (OR, 1.02; 95% CI, 0.99-1.04; *P* = .21).

**Conclusions and Relevance:**

This study found substantial and increasing disparities in CVD prevalence between the richest and poorest participants in the NHANES from 1999 to 2016, with lower CVD rate reported among the highest-resources group. Additional research into the dynamics of income inequality and health outcomes as well as policy and public health efforts to mitigate this inequality are needed.

## Key Points

**Question:**

How did the burden of cardiovascular disease in the United States differ between persons with the most resources (the top 20% of earners) and the rest of the population between 1999 and 2016?

**Findings:**

In this serial cross-sectional analysis of a nationally representative sample of 44 986 participants, decreases in cardiovascular disease prevalence primarily occurred in the highest-resources group, whereas the prevalence among the rest of the population declined at a much lower rate, stayed the same, or increased, depending on the cardiovascular condition.

**Meaning:**

Findings of this study suggest that substantial and increasing disparities in cardiovascular disease prevalence exist in the United States between people in the highest-resources group and the remainder of the population; further research into the drivers of such disparities is needed as well as policy and public health efforts to mitigate the consequences of these inequality dynamics.

## Teaser

This cross-sectional study examines the association between socioeconomic status and self-reported prevalence of congestive heart failure, angina, stroke, and heart attack among adult participants in the National Health and Nutrition Examination Survey.

## Introduction

Income inequality has increased dramatically over the past few decades in the United States, reaching levels the country has not experienced since the Great Depression.^1^ The growth of income inequality has not gone unnoticed, and substantial public conversation has ensued about the causes and potential consequences of the income gap. However, much of the conversation has focused on the gains of the top 1% of income earners, which overlooks the widening gap between the richest 20% of the people in the United States (those with the highest resources) and the poorest 80% of the population (the remainder of the population) that may have several implications for people and their health.

Between 1979 and 2013, the income of the richest people grew by $4 trillion, a trillion more than the income earned by the remaining 80% of the population.^2^ Over the past 2 decades, the income, adjusted for inflation, of the richest group has increased, whereas the income of the poorest people has either stayed constant or decreased.^3^ In 2018, the mean annual income for the richest people was approximately $234 000 per household and $111 379 per earner compared with a mean annual income of approximately $54 053 per household and $45 258 per earner for the poorest 80%.^4^ In addition, the richest people have access to a number of other demographic assets that distinguish them from the rest of the population. For example, most people in the highest income group are married (77%) and more than two-thirds (67%) have a college degree or higher educational level. Conversely, a smaller proportion of people in the remainder of the population are married (41%) and fewer than one-third (28%) have a college degree or higher educational level.^4^

The association between income and health is unequivocal; persons with higher income have lower morbidity and mortality across nearly all health indicators, longevity and mortality rates in particular.^5,6,7,8,9,10^ This association is likely driven principally by the salutary resources afforded by a higher income, such as access to health care, affordability of rising medication costs, better housing, and healthier food. As such, it is not surprising that health data suggest that the people with the most resources in the United States are accumulating more health, whereas those with the least resources are left behind in health care. Early evidence of this suggestion, for example, is the observation that life expectancy over the past several decades has been increasing consistently among the people in the highest income group, with far fewer, if any, gains seen among people in the remainder of the population.^11^

We hypothesized, therefore, that the widening income gap between the 2 groups (the highest-resources group and the remainder of the population) may be an important contributor to the dynamics of health inequality in the United States during the first quarter of the 21st century.^12^ In particular, the worsening population-level health indicators^13^ may be disproportionately attributed to a widening gap in health outcomes between those with the most resources and the rest of the population. To explore this hypothesis, we used a nationally representative data set to assess the trends in cardiovascular disease (CVD) in the 2 groups over the past 2 decades. We focused on CVD because of its primary role in the morbidity and mortality rates in the United States. Since the 1960s, the rapid decline in cardiovascular mortality has been associated with improved life expectancy.^14,15^ However, evidence shows that this decrease is slowing down or even reversing.^13,16^

This serial cross-sectional study aimed (1) to quantify the contribution of people in the highest-resources group and the remainder of the population to the burden of CVD and (2) to estimate the trends in the prevalence of CVD for the 2 groups from 1999 to 2016.

## Methods

### Data Source and Study Population

We used the National Health and Nutrition Examination Survey (NHANES) deidentified and publicly available data set to assess the prevalence of and trends in CVD on the basis of income level.^17^ Data collection for the NHANES was approved by the National Center for Health Statistics (NCHS) Research Ethics Review Board, with the requirement of documented consent from all participants.^18^ The Boston University Medical Campus Ethical Board deemed this study exempt from review according to the Common Rule because publicly available data sets were used. We followed the Strengthening the Reporting of Observational Studies in Epidemiology (STROBE) reporting guideline.

This cross-sectional analysis included data from 9 waves of the NHANES between 1999 and 2016. To produce estimates with greater precision and smaller sampling error, we constructed weights for the 9 survey cycles of combined data from 1999 to 2016 using the NHANES guidelines.^19^ Specifically, we used 4-year weights for survey cycles 1 and 2 (1999-2002) and 2-year weights for survey cycles 3 to 9 (2003-2016) to create an 18-year weight variable that represents the study population between 1999 and 2016. Demographic variables included in this analysis were participant age, sex, race/ethnicity, marital status, educational level, and US citizenship status.^20^ In addition, we included medical conditions that are CVD risk factors, such as obesity and high systolic blood pressure (SBP) reported in the medical examination section of the NHANES. Obesity was defined as a body mass index (calculated as weight in kilograms divided by height in meters squared) of 30 or greater. Mean SBP was calculated from 3 consecutive readings, with a fourth measurement taken if a previous one was incomplete. High SBP was defined as a mean SBP of 130 mm Hg or higher.

### Income Variables

To perform an accurate estimation of participant financial status, we used income to poverty ratio rather than annual household income to divide the population into 2 groups (highest resources with a ≥5 ratio, and the remainder of the population with a <5 ratio). The income to poverty ratio is an index developed by the NHANES that represents the annual family income adjusted for family size and the poverty threshold guidelines developed by the US Department of Health and Human Services, which depend on costs of living in a particular geographic location.^21^ Income to poverty ratio data were not available for participants who reported their income as less than $20 000 or more than $20 000 without specifying their exact income bracket and for participants who had missing annual income data. Participants with missing ratio data were excluded from this analysis. We converted the ratio to a binary variable of either 5 or greater (for the highest-resources group) or less than 5 (for the remainder of the population). We aimed to create a cutoff point that would divide the US population into the richest 20% of people and the poorest 80% of people, to test our hypothesis that, in this country, the 20% to 80% divide in income over the past few decades may also be present in trends of CVD burden, especially given that income is independently associated with CVD.

We attempted to examine differences in CVD prevalence between the richest and poorest of the participants. However, the analysis distribution did not fully align with the 20% to 80% cutoff because the NHANES coded participants with an income to poverty ratio of 5 or greater in 1 category to address potential confidentially issues and disclosure concerns, without which the deidentification of the subset of participants with the most resources will be challenging. Because of this NHANES policy, the highest-resources group represented between 22% and 26% (weighted) of the sample in a survey cycle in the present study.

### Cardiovascular Disease Variables

Outcomes included the self-reported diagnosis of congestive heart failure (CHF), angina, heart attack, or stroke among those 20 years or older, reflecting the age group for which the NHANES begins to collect data on CVD for adults. The diagnosis was obtained in the NHANES through asking participants whether they “were told by a healthcare provider” about one of the CVD outcomes.

### Statistical Analysis

We performed a descriptive analysis of demographic and medical characteristics using SAS FREQ procedure (SAS Institute Inc). We compared the distribution of these characteristics among participants in the highest-resources group vs the remainder of the population using a χ^2^ test.

Next, we used the 2010 census estimates to calculate the age-standardized prevalence of CVD in 3 age groups: 20 to 39 years, 40 to 59 years, and 60 years or older. Estimation was carried out with SAS SURVEYREG procedure, which accounted for the complex NHANES design by using the 18-year sample weight variable that we constructed. To examine the difference in prevalence by income group over time, we performed subgroup analyses that stratified income level using the SAS DOMAIN statement. To identify the trends in CVD prevalence over 9 consecutive survey cycles, we calculated the age-standardized prevalence of each of the outcomes through linear regression (SURVEYREG procedure), with survey cycle as a continuous variable. The magnitude of trends was assessed using the point estimates of survey cycle variable.

We applied SAS SURVEYLOGISTIC regression models to control for potential confounders. In the first model, we controlled for demographic variables such as age, sex, race/ethnicity, marital status, and US citizenship status. In the second model, we controlled for demographic variables as well as CVD risk factors (obesity and high SBP) and created interactions between the dichotomized income to poverty ratio variable and the educational level to account for a well-established association between income and different educational levels.^22^ We included interaction variables between survey cycle and the binary income to poverty ratio variable in both models to further assess the association between income group and the CVD prevalence trend during the study period.

For these analyses, the statistical significance was set at 2-sided *P* < .05, and all tests were 2-sided. Data were analyzed in December 2019.

## Results

The population in this analysis included 44 986 participants 20 years or older with data on income to poverty ratio; this sample accounted for 90.9% of participants in the NHANES between 1999 and 2016. Among the 7926 participants in the highest-resources group, 4094 (51.9%) were men, 3832 (48.1%) were women, and 3290 (50.3%) were in the 40- to 59-year age group (Table). Among the 37 060 participants in the remainder of the population, 19 470 (53.2%) were women, 17 590 (46.8%) were men, and 10 840 (34.1%) were in the 40- to 59-year age group (Table). The overall prevalence of CHF was 3.4% (n = 1523), angina was 3.0% (n = 1347), heart attack was 4.4% (n = 1978), and stroke was 3.9% (n = 1742).

**Table.** Characteristics of Study Participants, 1999-2016^a^

Characteristic	No. (weighted %)		*P* value
	Family income to poverty ratio ≥5 (n = 7926)	Family income to poverty ratio <5 (n = 37 060)	
Age, y			<.001
20-39	2307 (28.3)	13 493 (41.2)	
40-59	3290 (50.3)	10 840 (34.1)	
≥60	2329 (21.4)	12 727 (24.7)	
Sex			<.001
Men	4094 (51.9)	17 590 (46.8)	
Women	3832 (48.1)	19 470 (53.2)	
Race/ethnicity			<.001
Non-Hispanic White	5012 (84.2)	15 807 (64.5)	
Non-Hispanic Black	1183 (5.6)	8059 (12.9)	
Hispanic and Mexican	881 (4.1)	10 421 (16.1)	
Other	850 (6.1)	2773 (6.5)	
Marital status^b^			<.001
Not married	2374 (28.2)	18 642 (48.8)	
Married	5464 (71.8)	18 018 (51.2)	
Educational level^c^			<.001
Without high school diploma/GED certificate	417 (3.8)	11 936 (22.4)	
With high school diploma/GED certificate	1077 (13.7)	9269 (26.9)	
Some college or associate’s degree	2174 (27.1)	10 385 (32.2)	
College degree or higher	4256 (55.4)	5416 (18.5)	
Citizenship status^d^			<.001
US citizenship	7488 (96.5)	31 307 (89.7)	
Non-US citizenship	436 (3.5)	5700 (10.3)	
BMI^e^			<.001
≥30 (obesity)	2285 (29.2)	12 894 (36.5)	
<30 (no obesity)	5177 (70.8)	21 851 (63.5)	
SBP^f^			<.001
≥130 mm Hg (high)	1956 (25.1)	10 311(27.9)	
<130 mm Hg (not high)	4957 (74.9)	21 346 (72.1)	

Abbreviations: BMI, body mass index (calculated as weight in kilograms divided by height in meters squared); GED, General Educational Development; SBP, systolic blood pressure.

^a^ Data were from the National Health and Nutrition Examination Survey (NHANES) between 1999 and 2016 (N = 44 986). The highest-resources group cutoff was defined by income to poverty ratio of 5 or higher in the NHANES data sets.

^b^ Marital status was missing for 488 participants.

^c^ Educational level was missing for 56 participants.

^d^ Citizenship status was missing for 55 participants.

^e^ BMI was missing for 2779 participants.

^f^ SBP was missing for 6416 participants.

Major demographic differences between the 2 groups included marital status and educational levels. Most people in the highest-resources group were married (5464 [71.8%]) and had a college degree or higher (4256 [55.4%]). In the remainder of the population, slightly more than half of the participants (18 018 [51.2%]) were married and only a small proportion (5416 [18.5%]) had a college degree or higher. The 2 groups also differed in their race/ethnicity composition. In the highest-resources group, 5012 participants (84.2%) self-identified as White, 1183 (5.6%) as Black, and 881 (4.1%) as Hispanic and Mexican. In the remainder of the population, a smaller proportion of participants identified as White (15 807 [64.5%]), and a larger proportion identified as Black (8059 [12.9%]) or as Hispanic and Mexican (10 421 [16.1%]) (Table).

### Overall Age-Standardized CVD Prevalence by Income Group

**Figure 1.**
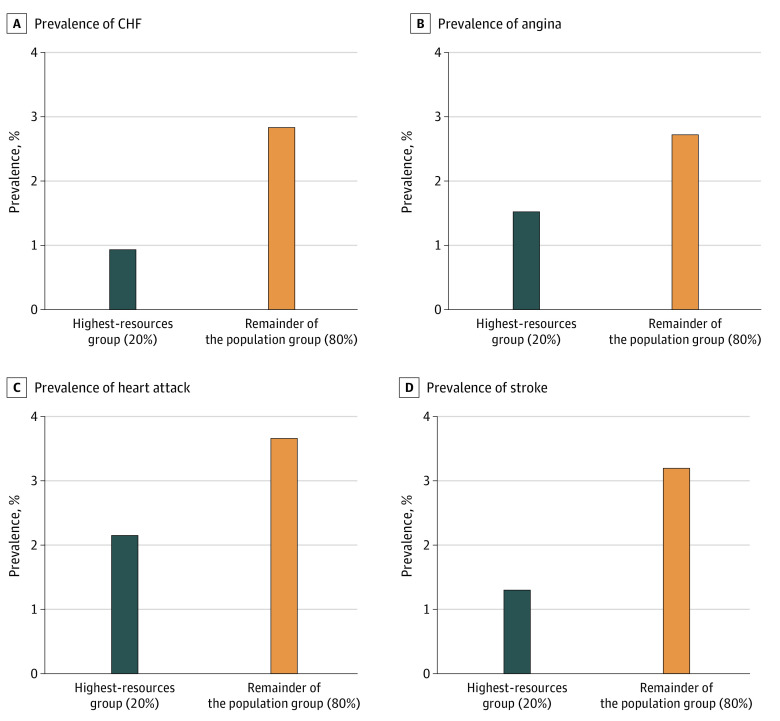
Overall Age-Standardized Prevalence of Cardiovascular Disease Among Participants 20 Years or Older Stratified by Income Group, 1999-2016 CHF indicates congestive heart failure.

Participants in the highest-resources group had a lower CVD prevalence than participants in the remainder of the population. The widest gap in age-standardized prevalence between the 2 groups was for stroke (1.3% [n = 146] vs 3.2% [n = 1596]) and CHF (0.9% [n = 121] vs 2.8% [n = 1402]), followed by heart attack (2.1% [n = 231] vs 3.7% [n = 1747]) and angina (1.5% [n = 166] vs 2.7% [n = 1181]) (Figure 1A-D).

### Trends in CVD Prevalence

**Figure 2.**
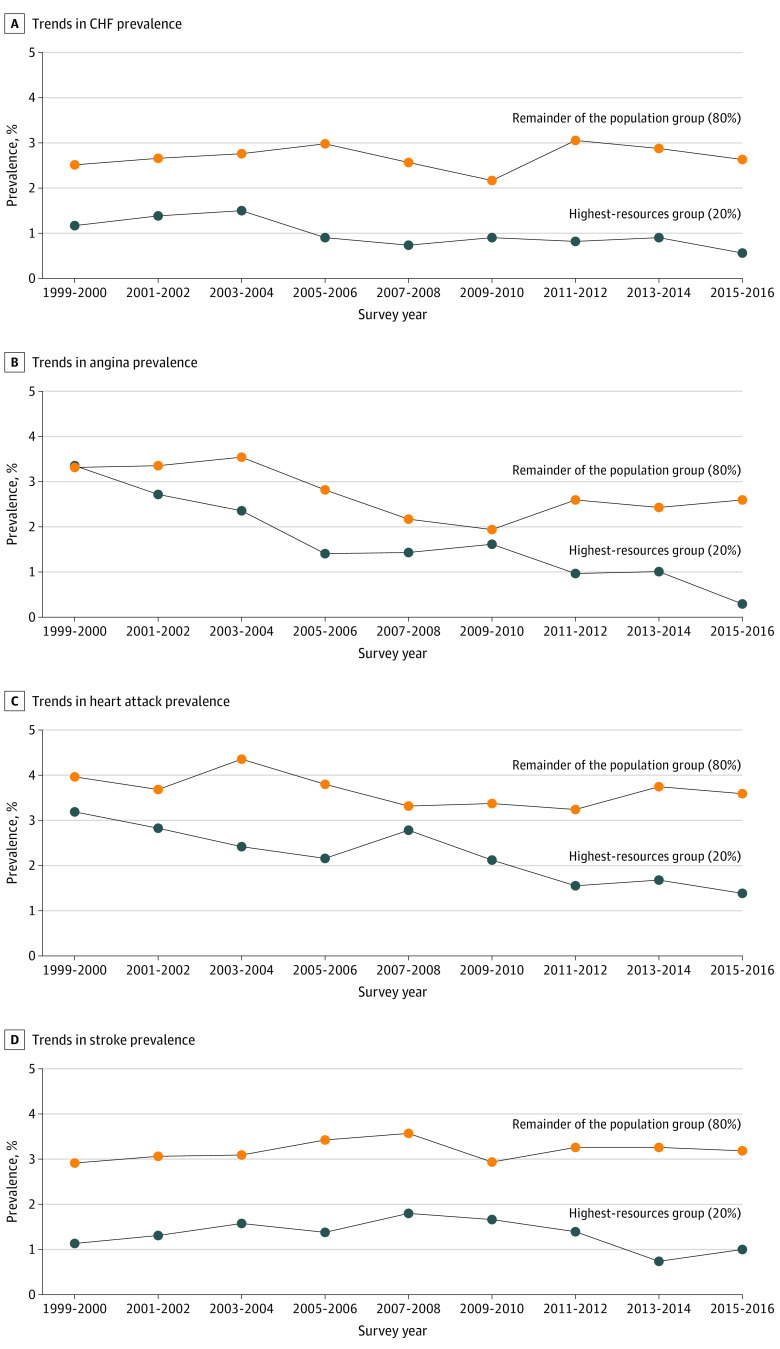
Age-Standardized Trends in Prevalence of Cardiovascular Disease Outcomes Among Participants 20 Years or Older Stratified by Income Group, 1999-2016 CHF indicates congestive heart failure.

**Figure 3.**
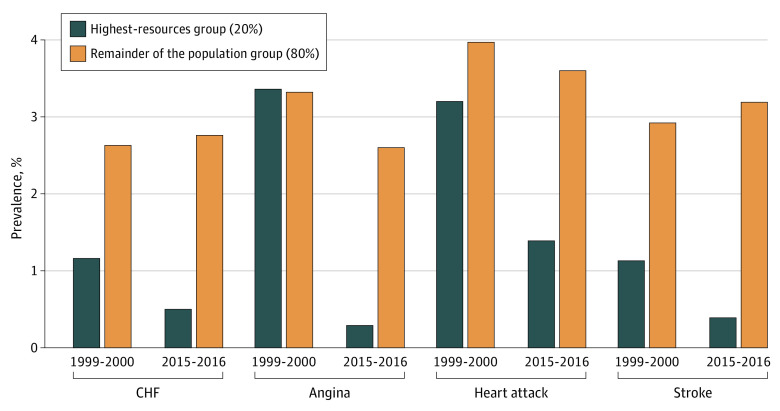
Comparison of Age-Standardized Prevalence in 1999-2000 vs 2015-2016, Stratified by Income Group CHF indicates congestive heart failure.

Among the highest-resources group, a decrease in the prevalence of CVD was observed between 1999 and 2016. The prevalence of CHF decreased from 1.2% (n = 11) in 1999 to 0.5% (n = 7) in 2016, an 11% reduction per survey cycle (point estimate, −0.11 [95% CI, −0.20 to −0.03]; *P* = .007); angina from 3.4% (n = 24) in 1999 to 0.3% (n = 5) in 2016, a 35% reduction per survey cycle (point estimate, −0.35 [95% CI, −0.49 to −0.21]; *P* < .001); heart attack from 3.2% (n = 24) in 1999 to 1.4% (n = 19) in 2016, a 24% reduction per survey cycle (point estimate, −0.24 [95% CI, −0.36 to −0.11]; *P* = .0003); and stroke from 1.1% (n = 8) in 1999 to 1.0% (n = 10) in 2016, a nonsignificant reduction of 5% per survey cycle (point estimate, −0.05 [95% CI, −0.15 to 0.04]; *P* = .29) (Figure 2A-D and Figure 3).

In the remainder of the population, the prevalence of angina decreased from 3.3% (n = 131) in 1999 to 2.6% (n = 118) in 2016, a 15% reduction per survey cycle (point estimate, −0.15 [95% CI, −0.24 to −0.05]; *P* = .002), and heart attack prevalence decreased from 4.0% (n = 160) in 1999 to 3.6% (n = 201) in 2016, a nonsignificant reduction of 7% per survey cycle (point estimate, −0.07 [95% CI, −0.16 to 0.02]; *P* = .15). Conversely, the prevalence of CHF increased from 2.6% (n = 123) in 1999 to 2.8% (n = 176) in 2016, a nonsignificant increase of 2% per survey cycle (point estimate, 0.02 [95% CI, −0.05 to 0.09]; *P* = .58), and stroke prevalence increased from 2.9% (n = 152) in 1999 to 3.2% (n = 178) in 2016, a nonsignificant increase of 2% per survey cycle (point estimate, −0.07 [95% CI, −0.16 to 0.02]; *P* = .64) (Figure 2A-D and Figure 3).

### Trends in the Association Between Income Group and CVD

Over time, decreased odds of reporting CVD were observed among those in the highest-resources group, when the model was adjusted for demographic variables. Specifically, the richest participants had lower odds of reporting CHF (odds ratio [OR], 0.90; 95% CI, 0.82-0.99; *P* = .03), angina (OR, 0.80; 95% CI, 0.73-0.87; *P* < .001), and heart attack (OR, 0.91; 95% CI, 0.86-0.97; *P* = .003), while there was no significant change in the odds of reporting stroke (OR, 0.97; 95% CI, 0.90-1.05; *P* = .43) (eTables 1-4 in the Supplement). When cardiovascular risk factors and the interaction between educational level and income were included in the model, over time, the highest-resources group continued to have lower odds of reporting angina (OR, 0.79; 95% CI, 0.72-0.86; *P* < .001) and heart attack (OR, 0.91; 95% CI, 0.85-0.96; *P* = .002), but there was no statistically significant change in the odds of reporting CHF (OR, 0.91; 95% CI, 0.81-1.01; *P* = .07) and stroke (OR, 0.98; 95% CI, 0.89-1.06; *P* = .58) (eTables 5-8 in the Supplement).

Over time, those in the remainder of the population had lower odds of reporting angina (OR, 0.95; 95% CI, 0.92-0.99; *P* = .007). The odds of reporting heart attack (OR, 0.99; 95% CI, 0.97-1.02; *P* = .06) were lower, but the difference was not statistically significant. Conversely, these participants had higher odds of reporting CHF (OR, 1.02; 95% CI, 1.00-1.05; *P* = .08) and stroke (OR, 1.02; 95% CI, 0.99-1.04; *P* = .21), but this difference was not statistically significant (eTables 1-4 in the Supplement). When cardiovascular risk factors and the interaction between educational level and income were included in the model, over time, the poorest participants had lower odds of reporting angina (OR, 0.95; 95% CI, 0.91-0.98; *P* = .005). Conversely, they had higher odds of reporting stroke (OR, 1.03; 95% CI, 1.00-1.07; *P* = .04). Althought the odds of reporting CHF (OR, 1.03; 95% CI, 1.00-1.06; *P* = .07) heart attack (OR, 1.00; 95% CI, 0.97-1.03; *P* = .96) were higher, the difference was not statistically significant (eTables 5-8 in the Supplement).

The interaction between the differences in trends in the 2 groups was statistically significant in both logistic regression analyses models. In the first model, the interaction was statistically significant for CHF (SE, 0.05; *P* = .01), angina (SE, 0.04; *P* < .001), and heart attack (SE, 0.03; *P* = .01) but not for stroke (SE, 0.04; *P* = .26). In the second model, the interaction was statistically significant for CHF (SE, 0.06; *P* = .03), angina (SE, 0.05; *P* = .0002), and heart attack (SE, 0.04; *P* = .006) but not for stroke (SE, 0.05; *P* = .22).

### Association Between Other Variables and CVD

Both of the logistic regression analyses models showed that, overall, older age was associated with increased odds of reporting CVD. Compared with the youngest age group (20-39 years), the ORs of CVD ranged from 4.41 (95% CI, 3.12-6.23) to 9.96 (95% CI, 6.95-14.27) for participants aged 40 to 59 years and from 15.19 (95% CI, 11.15-20.68) to 37.36 (95% CI, 26.45-52.78) for people 60 years or older. In contrast, women mainly had lower odds of reporting CVD than men (OR ranged from 0.40 [95% CI, 0.35-0.47] to 0.70 [95% CI, 0.61-0.81]), except for stroke (model 1: OR, 1.02 [95% CI, 0.89-1.18]; model 2: OR, 1.03 [95% CI, 0.86-1.25]). Married vs nonmarried participants had lower odds of reporting CVD (OR ranged from 0.73 [95% CI, 0.63-0.84] to 0.91 [95% CI, 0.77-1.08]), and those without US citizenship had lower odds of reporting CVD compared with those with US citizenship (OR ranged from 0.44 [95% CI, 0.29-0.68] to 0.61 [95% CI, 0.42-0.89]) (eTables 1-8 in the Supplement).

An inverse association was found between educational level and the odds of reporting CVD in the first model. Compared with those without a high school diploma or General Educational Development (GED) certificate, those with a high school diploma or GED certificate (OR ranged from 0.63 [95% CI, 0.52-0.76] to 0.76 [95% CI, 0.62-0.92]) followed by those with some college education (OR ranged from 0.59 [95% CI, 0.49-0.72] to 0.83 [95% CI, 0.66-1.04]) had the lowest protective odds against CVD. The most protective odds were for those with a college degree or higher (OR ranged from 0.39 [95% CI, 0.29-0.51] to 0.58 [95% CI, 0.46-0.72]) (eTables 1-4 in the Supplement). When the educational level and income interaction was included in the second model, more educational attainment generally showed lower odds of reporting CVD for the 2 groups.

**Figure 4.**
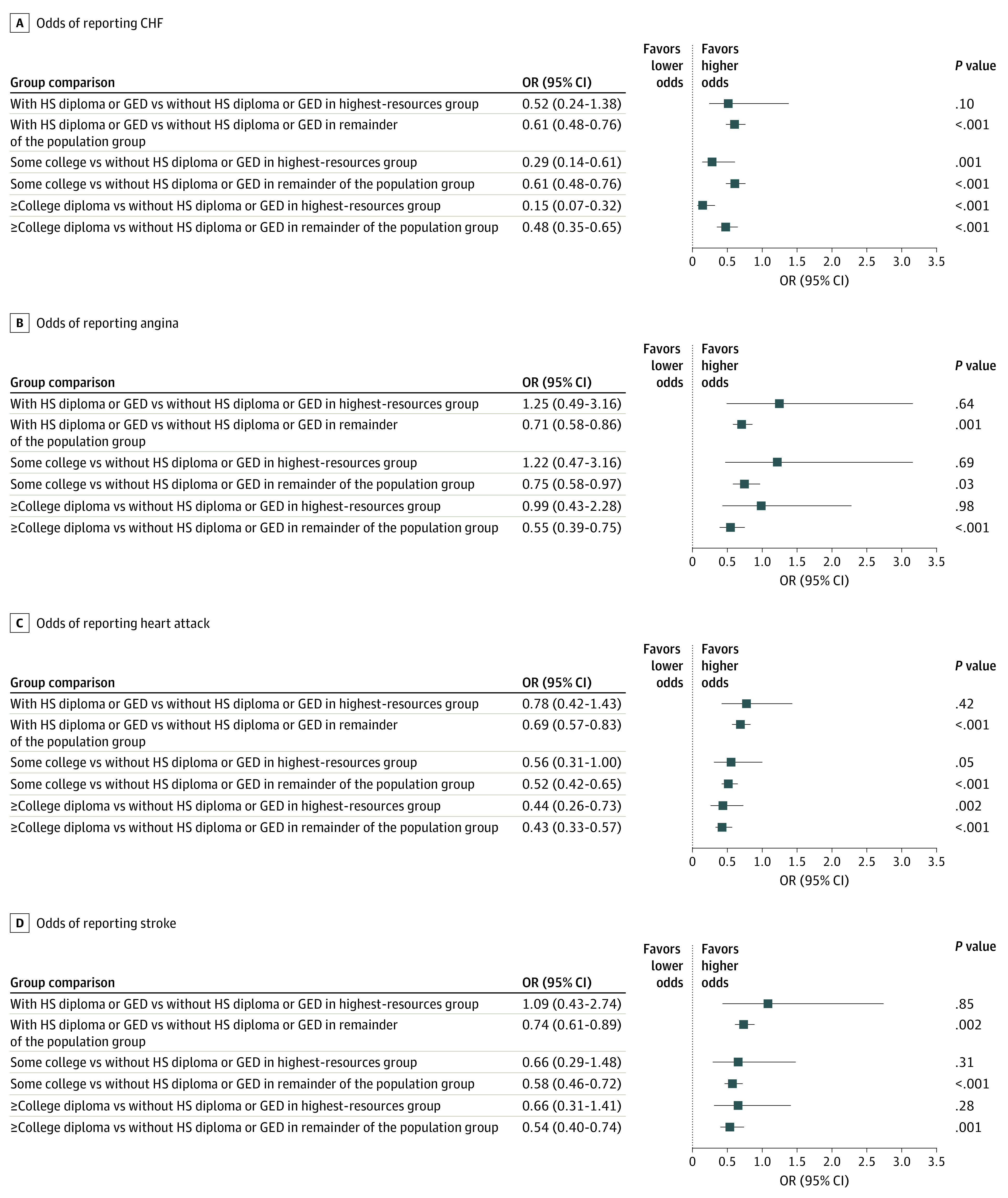
Odds Ratios of Cardiovascular Disease by Educational Level and Income Group, 1999-2016 CHF indicates congestive heart failure; GED, General Educational Development; HS, high school; OR, odds ratio.

Among the highest-resources group, people with a high school diploma or GED certificate, compared with those without, had lower odds of reporting CHF (OR, 0.52; 95% CI, 0.24-1.38) and heart attack (OR, 0.78; 95% CI, 0.42-1.43) and higher odds of reporting angina (OR, 1.25; 95% CI, 0.49-3.16) and stroke (OR, 1.09; 95% CI, 0.43-2.74) (Figure 4A-D). People with some college education vs those without a high school diploma or GED certificate had lower odds of reporting CVD (OR ranged from 0.29 [95% CI, 0.14-0.61] to 0.66 [95% CI, 0.29-1.48]), except for angina (OR, 1.22; 95% CI, 0.47-3.16). The most protective odds were for those with a college degree or higher (OR ranged from 0.15 [95% CI, 0.07-0.32] to 0.99 [95% CI, 0.43-2.28]), compared with those without a high school diploma or GED certificate (Figure 4A-D; eTables 5-8 in the Supplement).

Among people in the remainder of the population, the lowest protective odds against CVD were for those with a high school diploma or GED certificate (OR ranged from 0.61 [95% CI, 0.48-0.76] to 0.74 [95% CI, 0.61-0.89]) followed by some college education (OR ranged from 0.52 [95% CI, 0.42-0.65] to 0.75 [95% CI, 0.58-0.97]), compared with those without such educational attainment. The most protective odds were for those with a college degree or higher (OR ranged from 0.43 [95% CI, 0.33-0.57] to 0.55 [95% CI, 0.39-0.75]) (Figure 4A-D).

The association between race/ethnicity and CVD was mixed in the first model, which included only demographic variables. Compared with Black participants, White participants had lower odds of reporting CHF (OR, 0.83; 95% CI, 0.72-0.97) and stroke (OR, 0.80; 95% CI, 0.70-0.93) and higher odds of reporting angina (OR, 1.71; 95% CI, 1.42-2.07) and heart attack (OR, 1.32; 95% CI, 1.14-1.54) (eTables 1-4 in the Supplement). The second model that included cardiovascular risk factors and the educational level and income interaction outputted similar but more pronounced results. Overall, Hispanic and Mexican participants compared with Black participants had lower odds of reporting CVD (OR ranged from 0.58 [95% CI, 0.45-0.75] to 0.85 [95% CI, 0.68-1.06]) but not angina (model 1: OR, 1.27 [95% CI, 0.99-1.62]; model 2: OR, 1.26 [95% CI, 0.95-1.68]) (eTables 1-8 in the Supplement).

## Discussion

Using data from a nationally representative sample of the US population between 1999 and 2016, we found a substantially lower burden of CVD among the people with the most resources compared with the remainder of the population. The overall prevalence of CHF was less than one-third (0.9% vs 2.8%) and that of stroke was less than one-half (from 1.3% to 3.2%) in the highest-resources group compared with the remainder of the population. In addition, disparities in CVD between the 2 groups widened between 1999 and 2016. This gap was most evident in the prevalence of angina, which decreased about 5-fold among the highest-resources group compared with the remainder of the population during this period. When controlled for demographic variables, the model showed that those in the highest-resources group had up to 20% lower odds of reporting CVD in 2016 than in 1999. In contrast, the remainder of the population had higher odds of reporting both CHF and stroke.

Numerous studies support the role of socioeconomic status in shaping CVD morbidity and mortality.^23,24,25,26,27^ Specifically, data from several studies suggest that income level is independently associated with CVD.^28,29,30,31,32^ The present study illuminates the dynamics of income differences in CVD in the United States. Previous studies have shown that the prevalence of angina and heart attack has been declining.^33,34^ Results of this study suggest that these trends in angina and heart attack are responsive to the decrease in prevalence among the richest NHANES participants. Conversely, studies show that the prevalence of CHF and stroke has been increasing and is projected to further increase during the next decade.^35,36^ Results of this study suggest that these trends in CHF and stroke are responsive to the increase in prevalence among 80% of the NHANES participants, whereas the prevalence among the richest group either remains constant or is decreasing. These 2 conditions (CHF and stroke) are associated with high out-of-pocket expenses, which are more burdensome for those in the remainder of the population.^37,38^ These 2 conditions are also major factors in US health care expenditures, suggesting that these widening disparities in prevalence are associated with the ever-greater cost in the health care system.^36,37,38,39^

We believe that this study recasts the understanding of the differences in CVD prevalence as one driven by the differences between persons in the richest quantile whose income and assets continue to increase and persons in the rest of the population whose socioeconomic achievements are stagnant. This viewpoint contrasts with the dominant approach of comparing those in the top 1% of the economy with everyone else. A substantially different architecture of inequality, one that points to other solutions, is needed. Policy and public health efforts should be directed to mitigate the consequences of these inequality dynamics.

### Limitations

This study has several limitations. First, the outcomes that were assessed relied on self-reported information. However, previous analyses suggest that self-reported outcomes in the NHANES are a valid tool for assessing prevalence.^40^ Second, the NHANES data structure limited our ability to create a clear 20 to 80 income cutoff. As such, the final results do not fully align with the 20 to 80 cutoff. In addition, not enough data points were available to enable us to conduct subanalyses that stratified the CVD prevalence by income group and by racial/ethnic group. As such, we could not report on whether the trend between income and CVD prevalence is different for minority groups. Third, we used serial cross-sectional data. Further research is needed to establish the causal relationships between income and CVD in the United States.

## Conclusions

This cross-sectional study found substantial and increasing disparities in CVD between people with the most resources and the remainder of the US population. Over the past 2 decades, decreases in CVD prevalence primarily occurred in the highest-resources group, whereas the prevalence among the remainder of the population declined at a much lower rate, stayed the same, or increased, depending on the specific cardiovascular condition. Moreover, the largest disparities in CVD were in conditions associated with high out-of-pocket and health care expenditures. These findings should motivate further research into the dynamics of income inequality and health outcomes as well as the potential mechanisms behind these inequalities, such as increasing health care expenditures, behavioral risk factors, or other structural factors, which can point to potential solutions. Mitigating the consequences of these inequality dynamics requires the development of policy and public health efforts.

## Notes

Supplement. **eTable 1.** Model 1: Multivariable Analysis of the Association Between Congestive Heart Failure and Demographics, 1999-2016 **eTable 2.** Model 1: Multivariable Analysis of the Association Between Angina and Demographics, 1999-2016 **eTable 3.** Model 1: Multivariable Analysis of the Association Between Heart Attack and Demographics, 1999-2016 **eTable 4.** Model 1: Multivariable Analysis of the Association Between Stroke and Demographics, 1999-2016 **eTable 5.** Model 2: Multivariable Analysis of the Association Between Congestive Heart Failure, Demographics, and CVD Risk Factors, 1999-2016 **eTable 6.** Model 2: Multivariable Analysis of the Association Between Angina, Demographics, and CVD Risk Factors, 1999-2016 **eTable 7.** Model 2: Multivariable Analysis of the Association Between Heart Attack, Demographics, and CVD Risk Factors, 1999-2016 **eTable 8.** Model 2: Multivariable Analysis of the Association Between Stroke, Demographics, and CVD Risk Factors, 1999-2016
