# Supplementary material for: Trends in Cardiovascular Disease Prevalence by Income Level in the United States
Source: JAMA Netw Open. 2020 Sep 25;3(9):e2018150. doi: 10.1001/jamanetworkopen.2020.18150 (PMC7519418; doi:10.1001/jamanetworkopen.2020.18150)
Supplement: Supplement. — eTable 1. Model 1: Multivariable Analysis of the Association Between Congestive Heart Failure and Demographics, 1999-2016 eTable 2. Model 1: Multivariable Analysis of the Association Between Angina and Demographics, 1999-2016 eTable 3. Model 1: Multivariable Analysis of the Association Between Heart Attack and Demographics, 1999-2016 eTable 4. Model 1: Multivariable Analysis of the Association Between Stroke and Demographics, 1999-2016 eTable 5. Model 2: Multivariable Analysis of the Association Between Congestive Heart Failure, Demographics, and CVD Risk Factors, 1999-2016 eTable 6. Model 2: Multivariable Analysis of the Association Between Angina, Demographics, and CVD Risk Factors, 1999-2016 eTable 7. Model 2: Multivariable Analysis of the Association Between Heart Attack, Demographics, and CVD Risk Factors, 1999-2016 eTable 8. Model 2: Multivariable Analysis of the Association Between Stroke, Demographics, and CVD Risk Factors, 1999-2016 [file jamanetwopen-e2018150-s001.pdf]

# Supplementary Online Content

Abdalla SM, Yu S, Galea S. Trends in cardiovascular disease prevalence by income level in the United States. *JAMA Netw Open*. 2020;3(9):e2018150.
doi:10.1001/jamanetworkopen.2020.18150

**eTable 1.** Model 1: Multivariable Analysis of the Association Between Congestive Heart Failure and Demographics, 1999-2016
**eTable 2.** Model 1: Multivariable Analysis of the Association Between Angina and Demographics, 1999-2016
**eTable 3.** Model 1: Multivariable Analysis of the Association Between Heart Attack and Demographics, 1999-2016
**eTable 4.** Model 1: Multivariable Analysis of the Association Between Stroke and Demographics, 1999-2016
**eTable 5.** Model 2: Multivariable Analysis of the Association Between Congestive Heart Failure, Demographics, and CVD Risk Factors, 1999-2016
**eTable 6.** Model 2: Multivariable Analysis of the Association Between Angina, Demographics, and CVD Risk Factors, 1999-2016
**eTable 7.** Model 2: Multivariable Analysis of the Association Between Heart Attack, Demographics, and CVD Risk Factors, 1999-2016
**eTable 8.** Model 2: Multivariable Analysis of the Association Between Stroke, Demographics, and CVD Risk Factors, 1999-2016

This supplementary material has been provided by the authors to give readers additional information about their work.

**eTable 1.** Model 1: Multivariable Analysis of the Association Between Congestive Heart Failure and Demographics, 1999-2016

| Variables | Odds ratio (95% CI) | *p*-value |
|---|---|---|
| **Age** | | |
| 40-59 vs. 20-39 | 6.70 (4.64, 9.66) | <0.0001 |
| 60+ vs. 20-39 | 28.42 (19.12, 42.24) | <0.0001 |
| **Gender** | | |
| female vs. male | 0.69 (0.61, 0.79) | <0.0001 |
| **Race/Ethnicity** | | |
| White vs. Black | 0.83 (0.72, 0.97) | 0.02 |
| Hispanic/Mexican vs. Black | 0.58 (0.45, 0.75) | <0.0001 |
| Other vs. Black | 0.82 (0.56, 1.20) | 0.31 |
| **Marital status** | | |
| married vs. not married | 0.74 (0.65, 0.86) | <0.0001 |
| **Education** | | |
| High school graduate vs. no high school diploma | 0.63 (0.52, 0.76) | <0.0001 |
| Some college vs. no high school diploma | 0.62 (0.51, 0.75) | <0.0001 |
| College graduate or above vs. no high school diploma | 0.39 (0.29, 0.51) | <0.0001 |
| **Citizenship status** | | |
| Non-US citizen vs. US citizen | 0.55 (0.39, 0.76) | 0.0004 |
| **Survey cycle and income group interaction** | | |
| Trend among richest 20% | 0.9 (0.82, 0.99) | 0.03 |
| Trend among poorest 80% | 1.02 (1.00, 1.05) | 0.08 |

Data are from the National Health and Nutrition Examination Survey (NHANES) between 1999-2016 (N=44,986). Congestive heart failure data missing for 132 participants. Marital status missing from 488 participants, education missing from 56 participants, citizenship status missing from 55 participants. The Richest 20% cut-off was defined by income-to-poverty $\geq$ 5 in NHANES datasets.

**eTable 2.** Model 1: Multivariable Analysis of the Association Between Angina and Demographics, 1999-2016

| Variables | Odds ratio (95% CI) | *p*-value |
|---|---:|---:|
| **Age** | | |
| 40-59 vs. 20-39 | 8.19 (5.72, 11.73) | <0.0001 |
| 60+ vs. 20-39 | 26.23 (18.02, 38.17) | <0.0001 |
| **Gender** | | |
| female vs. male | 0.70 (0.61, 0.81) | <0.0001 |
| **Race/Ethnicity** | | |
| White vs. Black | 1.71 (1.42, 2.07) | <0.0001 |
| Hispanic/Mexican vs. Black | 1.27 (0.99, 1.62) | 0.06 |
| Other vs. Black | 1.49 (1.01, 2.20) | 0.04 |
| **Marital status** | | |
| married vs. not married | 0.91 (0.77, 1.08) | 0.27 |
| **Education** | | |
| High school graduate vs. no high school diploma | 0.76 (0.62, 0.92) | 0.0053 |
| Some college vs. no high school diploma | 0.83 (0.66, 1.04) | 0.1016 |
| College graduate or above vs. no high school diploma | 0.58 (0.46, 0.72) | <0.0001 |
| **Citizenship status** | | |
| Non-US citizen vs. US citizen | 0.45 (0.32, 0.65) | <0.0001 |
| **Survey cycle and income group interaction** | | |
| Trend among richest 20% | 0.80 (0.73, 0.87) | <0.0001 |
| Trend among poorest 80% | 0.95 (0.92, 0.99) | 0.0072 |

Data are from the National Health and Nutrition Examination Survey (NHANES) between 1999-2016 (N=44,986). Angina data missing for 145 participants. Marital status missing from 488 participants, education missing from 56 participants, citizenship status missing from 55 participants. The Richest 20% cut-off was defined by income-to-poverty ≥ 5 in NHANES datasets.

©2020 Abdalla SM et al. *JAMA Network Open*

**eTable 3.** Model 1: Multivariable Analysis of the Association Between Heart Attack and Demographics, 1999-2016

| **Variables** | **Odds ratio (95% CI)** | ***p*-value** |
|---|---|---|
| **Age** | | |
| 40-59 vs. 20-39 | 9.13 (6.62, 12.58) | <0.0001 |
| 60+ vs. 20-39 | 33.06 (24.20, 45.18) | <0.0001 |
| **Gender** | | |
| female vs. male | 0.44 (0.38, 0.50) | <0.0001 |
| **Race/Ethnicity** | | |
| White vs. Black | 1.32 (1.14, 1.54) | 0.0003 |
| Hispanic/Mexican vs. Black | 0.79 (0.64, 0.97) | 0.03 |
| Other vs. Black | 1.48 (1.07, 2.04) | 0.02 |
| **Marital status** | | |
| married vs. not married | 0.74 (0.65, 0.85) | <0.0001 |
| **Education** | | |
| High school graduate vs. no high school diploma | 0.71 (0.61, 0.84) | <0.0001 |
| Some college vs. no high school diploma | 0.59 (0.51, 0.68) | <0.0001 |
| College graduate or above vs. no high school diploma | 0.44 (0.36, 0.53) | <0.0001 |
| **Citizenship status** | | |
| Non-US citizen vs. US citizen | 0.45 (0.28, 0.72) | 0.001 |
| **Survey cycle and income group interaction** | | |
| Trend among richest 20% | 0.91 (0.86, 0.97) | 0.0025 |
| Trend among poorest 80% | 0.99 (0.97, 1.02) | 0.063 |

Data are from the National Health and Nutrition Examination Survey (NHANES) between 1999-2016 (N=44,986). Heart attack data missing for 71 participants. Marital status missing from 488 participants, education missing from 56 participants, citizenship status missing from 55 participants. The Richest 20% cut-off was defined by income-to-poverty $\geq$ 5 in NHANES datasets.

©2020 Abdalla SM et al. *JAMA Network Open*

**eTable 4.** Model 1: Multivariable Analysis of the Association Between Stroke and Demographics, 1999-2016

| **Variables** | **Odds ratio (95% CI)** | ***p*-value** |
|---|---|---|
| **Age** | | |
| 40-59 vs. 20-39 | 4.93 (3.53, 6.89) | <0.0001 |
| 60+ vs. 20-39 | 18.08 (13.58, 24.08) | <0.0001 |
| **Gender** | | |
| female vs. male | 1.02 (0.89, 1.18) | 0.75 |
| **Race/Ethnicity** | | |
| White vs. Black | 0.81 (0.70, 0.93) | 0.004 |
| Hispanic/Mexican vs. Black | 0.61 (0.49, 0.76) | |
| Other vs. Black | 1.10 (0.83, 1.47) | <0.0001 |
| **Marital status** | | |
| married vs. not married | 0.73 (0.63, 0.84) | <0.0001 |
| **Education** | | |
| High school graduate vs. no high school diploma | 0.76 (0.65, 0.88) | 0.0003 |
| Some college vs. no high school diploma | 0.59 (0.49, 0.72) | <0.0001 |
| College graduate or above vs. no high school diploma | 0.55 (0.44, 0.69) | <0.0001 |
| **Citizen status** | | |
| Non-US citizen vs. US citizen | 0.49 (0.33, 0.72) | 0.0004 |
| **Survey cycle and income group interaction** | | |
| Trend among richest 20% | 0.97 (0.90, 1.05) | 0.43 |
| Trend among poorest 80% | 1.02 (0.99, 1.04) | 0.21 |

Data are from the National Health and Nutrition Examination Survey (NHANES) between 1999-2016 (N=44,986). Stroke data missing for 57 participants. Marital status missing from 488 participants, education missing from 56 participants, citizenship status missing from 55 participants. The Richest 20% cut-off was defined by income-to-poverty $\geq$ 5 in NHANES datasets.

**eTable 5.** Model 2: Multivariable Analysis of the Association Between Congestive Heart Failure, Demographics, and CVD Risk Factors, 1999-2016

| **Variables** | **Odds ratio (95% CI)** | ***p*-value** |
|---|---|---|
| **Age** | | |
| 40-59 vs. 20-39 | 7.05 (4.64, 10.72) | <0.0001 |
| 60+ vs. 20-39 | 28.91 (18.51, 45.14) | <0.0001 |
| **Gender** | | |
| female vs. male | 0.64 (0.54, 0.74) | <0.0001 |
| **Race/Ethnicity** | | |
| White vs. Black | 0.83 (0.69, 1.00) | 0.05 |
| Hispanic/Mexican vs. Black | 0.60 (0.45, 0.81) | 0.0008 |
| Other vs. Black | 0.85 (0.54, 1.35) | 0.49 |
| **Marital status** | | |
| married vs. not married | 0.78 (0.66, 0.91) | 0.003 |
| **Citizenship status** | | |
| Non-US citizen vs. US citizen | 0.61 (0.42, 0.89) | 0.01 |
| **Body Mass Index (BMI)** | | |
| Obese vs. not obese | 1.86 (1.58, 2.21) | <0.0001 |
| **Systolic blood pressure (SBP)** | | |
| Has high SBP vs. does not have high SBP | 0.92 (0.79, 1.08) | 0.31 |
| **Education and income group interaction** | | |
| High school graduate vs. no high school diploma among the richest 20% | 0.52 (0.24, 1.38) | 0.10 |
| Some college vs. no high school diploma among the richest 20% | 0.29 (0.14, 0.61) | 0.001 |
| College graduate or above vs. no high school diploma among the richest 20% | 0.15 (0.07, 0.32) | <0.0001 |
| High school graduate vs. no high school diploma among poorest 80% | 0.61 (0.48, 0.76) | <0.0001 |
| Some college vs. no high school diploma among poorest 80% | 0.61 (0.48, 0.76) | <0.0001 |
| College graduate or above vs. no high school diploma among the poorest 80% | 0.48 (0.35, 0.65) | <0.0001 |
| **Survey cycle and income group interaction** | | |
| Trend among the richest 20% | 0.91 (0.81, 1.01) | 0.07 |
| Trend among the poorest 80% | 1.03 (1.00, 1.06) | 0.07 |

Data are from the National Health and Nutrition Examination Survey (NHANES) between 1999-2016 (N=44,986). Angina data missing for 145 participants. Marital status missing from 488 participants, education missing from 56 participants, citizenship status missing from 55 participants. BMI missing from 2,779 participants, and systolic blood pressure missing from 6,416 participants. Obesity was defined as BMI of greater than or equal to 30 kg/m$^2$. High SBP was defined as SBP greater than or equal to 130 mm Hg. The Richest 20% cut-off was defined by income-to-poverty$\geq$ 5 in NHANES datasets.

©2020 Abdalla SM et al. *JAMA Network Open*

**eTable 6.** Model 2: Multivariable Analysis of the Association Between Angina, Demographics, and CVD Risk Factors, 1999-2016

| Variables | Odds ratio (95% CI) | *p*-value |
|---|---|---|
| **Age** | | |
| 40-59 vs. 20-39 | 8.76 (5.74, 13.38) | <0.0001 |
| 60+ vs. 20-39 | 29.06 (18.94, 44.60) | <0.0001 |
| **Gender** | | |
| female vs. male | 0.66 (0.56, 0.78) | <0.0001 |
| **Race/Ethnicity** | | |
| White vs. Black | 1.59 (1.26, 1.99) | 0.0001 |
| Hispanic/Mexican vs. Black | 1.26 (0.95, 1.68) | 0.11 |
| Other vs. Black | 1.63 (1.06, 2.52) | 0.03 |
| **Marital status** | | |
| married vs. not married | 0.87 (0.70, 1.00) | 0.17 |
| **Citizenship status** | | |
| Non-US citizen vs. US citizen | 0.51 (0.34, 0.76) | 0.001 |
| **Body Mass Index (BMI)** | | |
| Obese vs. not obese | 1.78 (1.53, 2.07) | <0.0001 |
| **Systolic blood pressure (SBP)** | | |
| Has high SBP vs. does not have high SBP | 0.84 (0.70, 1.00) | 0.04 |
| **Education and income group interaction** | | |
| High school graduate vs. no high school diploma among the richest 20% | 1.25 (0.49, 3.16) | 0.64 |
| Some college vs. no high school diploma among the richest 20% | 1.22 (0.47, 3.16) | 0.69 |
| College graduate or above vs. no high school diploma among the richest 20% | 0.99 (0.43, 2.28) | 0.98 |
| High school graduate vs. no high school diploma among poorest 80% | 0.71 (0.58, 0.86) | 0.0008 |
| Some college vs. no high school diploma among poorest 80% | 0.75 (0.58, 0.97) | 0.03 |
| College graduate or above vs. no high school diploma among the poorest 80% | 0.55 (0.39, 0.75) | 0.0003 |
| **Survey cycle and income group interaction** | | |
| Trend among the richest 20% | 0.79 (0.72, 0.86) | <0.0001 |
| Trend among the poorest 80% | 0.95 (0.91, 0.98) | 0.005 |

Data are from the National Health and Nutrition Examination Survey (NHANES) between 1999-2016 (N=44,986). Angina data missing for 145 participants. Marital status missing from 488 participants, education missing from 56 participants, citizenship status missing from 55 participants. BMI missing from 2,779 participants, and systolic blood pressure missing from 6,416 participants. Obesity was defined as BMI of greater than or equal to 30 $kg/m^2$. High SBP was defined as SBP greater than or equal to 130 mm Hg. The Richest 20% cut-off was defined by income-to-poverty $\geq$ 5 in NHANES datasets.

©2020 Abdalla SM et al. *JAMA Network Open*

**eTable 7.** Model 2: Multivariable Analysis of the Association Between Heart Attack, Demographics, and CVD Risk Factors, 1999-2016

| Variables | Odds ratio (95% CI) | *p*-value |
|---|---|---|
| **Age** | | |
| 40-59 vs. 20-39 | 9.96 (6.95, 14.27) | <0.0001 |
| 60+ vs. 20-39 | 37.36 (26.45, 52.78) | <0.0001 |
| **Gender** | | |
| female vs. male | 0.40 (0.35, 0.47) | <0.0001 |
| **Race/Ethnicity** | | |
| White vs. Black | 1.28 (1.09, 1.52) | 0.004 |
| Hispanic/Mexican vs. Black | 0.85 (0.68, 1.06) | 0.15 |
| Other vs. Black | 1.83 (1.31, 2.57) | 0.0005 |
| **Marital status** | | |
| married vs. not married | 0.75 (0.64, 0.87) | 0.0002 |
| **Citizenship status** | | |
| Non-US citizen vs. US citizen | 0.44 (0.29, 0.68) | 0.0002 |
| **Body Mass Index (BMI)** | | |
| Obese vs. not obese | 1.57 (1.38, 1.78) | <0/0001 |
| **Systolic blood pressure (SBP)** | | |
| Has high SBP vs. does not have high SBP | 0.87 (0.76, 1.00) | 0.04 |
| **Education and income group interaction** | | |
| High school graduate vs. no high school diploma among the richest 20% | 0.78 (0.42, 1.43) | 0.42 |
| Some college vs. no high school diploma among the richest 20% | 0.56 (0.31, 1.00) | 0.05 |
| College graduate or above vs. no high school diploma among the richest 20% | 0.44 (0.26, 0.73) | 0.002 |
| High school graduate vs. no high school diploma among poorest 80% | 0.69 (0.57, 0.83) | <0.0001 |
| Some college vs. no high school diploma among poorest 80% | 0.52 (0.42, 0.65) | <0.0001 |
| College graduate or above vs. no high school diploma among the poorest 80% | 0.43 (0.33, 0. 57) | <0.0001 |
| **Survey cycle and income group interaction** | | |
| Trend among the richest 20% | 0.91 (0.85, 0.96) | 0.002 |
| Trend among the poorest 80% | 1.00 (0.97, 1.03) | 0.96 |

Data are from the National Health and Nutrition Examination Survey (NHANES) between 1999-2016 (N=44,986). Heart attack data missing for 71 participants. Marital status missing from 488 participants, education missing from 56 participants, citizenship status missing from 55 participants. BMI missing from 2,779 participants, and systolic blood pressure missing from 6,416 participants. Obesity was defined as BMI of greater than or equal to 30 $kg/m^2$. High SBP was defined as SBP greater than or equal to 130 mm Hg. The Richest 20% cut-off was defined by income-to-poverty $\geq$ 5 in NHANES datasets.

©2020 Abdalla SM et al. *JAMA Network Open*

**eTable 8.** Model 2: Multivariable Analysis of the Association Between Stroke, Demographics, and CVD Risk Factors, 1999-2016

| **Variables** | **Odds ratio (95% CI)** | ***p*-value** |
|---|---|---|
| **Age** | | |
| 40-59 vs. 20-39 | 4.41 (3.12, 6.23) | <0.0001 |
| 60+ vs. 20-39 | 15.19 (11.15, 20.68) | <0.0001 |
| **Gender** | | |
| female vs. male | 1. 03 (0.86, 1.25) | 0.74 |
| **Race/Ethnicity** | | |
| White vs. Black | 0.80 (0.68, 0.93) | 0.004 |
| Hispanic/Mexican vs. Black | 0.61 (0.48, 0.78) | 0.0001 |
| Other vs. Black | 1.20 (0.86, 1.66) | 0.28 |
| **Marital status** | | |
| married vs. not married | 0.76 (0.66, 0.89) | 0.0007 |
| **Citizenship status** | | |
| Non-US citizen vs. US citizen | 0.52 (0.32, 0.82) | 0.005 |
| **Body Mass Index (BMI)** | | |
| Obese vs. not obese | 1.25 (1.06, 1.47) | 0.008 |
| **Systolic blood pressure (SBP)** | | |
| Has high SBP vs. does not have high SBP | 1.04 (0.88, 1.22) | 0.64 |
| **Education and income group interaction** | | |
| High school graduate vs. no high school diploma among the richest 20% | 1.09 (0.43, 2.74) | 0.85 |
| Some college vs. no high school diploma among the richest 20% | 0.66 (0.29, 1.48) | 0.31 |
| College graduate or above vs. no high school diploma among the richest 20% | 0.66 (0.31, 1.41) | 0.28 |
| High school graduate vs. no high school diploma among poorest 80% | 0.74 (0.61, 0.89) | 0.002 |
| Some college vs. no high school diploma among poorest 80% | 0.58 (0.46, 0.72) | <0.0001 |
| College graduate or above vs. no high school diploma among the poorest 80% | 0.54 (0.40, 0.74) | 0.0001 |
| **Survey cycle and income group interaction** | | |
| Trend among the richest 20% | 0.98 (0.89, 1.06) | 0.58 |
| Trend among the poorest 80% | 1.03 (1.00, 1.07) | 0.04 |

Data are from the National Health and Nutrition Examination Survey (NHANES) between 1999-2016 (N=44,986). Stroke data missing for 57 participants. Marital status missing from 488 participants, education missing from 56 participants, citizenship status missing from 55 participants. BMI missing from 2,779 participants, and systolic blood pressure missing from 6,416 participants. Obesity was defined as BMI of greater than or equal to 30 $kg/m^2$. High SBP was defined as SBP greater than or equal to 130 mm Hg. The Richest 20% cut-off was defined by income-to-poverty $\geq$ 5 in NHANES datasets.

©2020 Abdalla SM et al. *JAMA Network Open*
